# The impact of contextual family risks on prisoners' children's behavioural outcomes and the potential protective role of family functioning moderators

**DOI:** 10.1080/17405629.2015.1050374

**Published:** 2015-06-22

**Authors:** Lucy Markson, Michael E. Lamb, Friedrich Lösel

**Affiliations:** ^a^ Department of Psychology, University of Cambridge, UK; ^b^ Institute of Criminology, University of Cambridge, UK; ^c^ University of Erlangen-Nuremberg, Germany

**Keywords:** children of prisoners, cumulative risk, family functioning protective factors, behavioural development, resilience

## Abstract

Research suggests that children of prisoners have an increased risk for behavioural and emotional problems. However, in a resilience approach, one should expect heterogeneous outcomes and thus apply a contextualized perspective. As this is rarely acknowledged in empirical research, the present study sought to fill this gap using data from the Fragile Families and Child Wellbeing study on 801 children of imprisoned fathers. We explored the extent to which cumulative family risks measured during the first year of life (e.g., poverty and mental health problems) predicted behavioural outcomes at age 9 and whether potentially protective aspects of family functioning moderated the impact of these risk factors. Cumulative risk significantly predicted behavioural outcomes, but the associations were weak. No strong evidence of moderation was found. At low risk, mother–child closeness moderated behavioural outcomes. There was also some evidence of moderation by accumulated protective factors. Potential implications for policy and practice and challenges for further research are discussed.

## Introduction

Parental imprisonment is a common phenomenon. In the USA, the nation with the highest rate of imprisonment in the world, 2.3% of children have an imprisoned parent at any one time (Glaze & Maruschak, 2008). African-American and socially excluded families are disproportionately affected (Wildeman, 2009). Much has been written about the negative impact of imprisonment on children, with the link to filial anti-social behaviour among the most robust associations after controlling for other factors (e.g., Murray, Farrington, & Sekol, 2012).

However, prisoners' children are not a homogenous group and there are many individual and environmental factors that may influence how they experience parental imprisonment. For example, a qualitative study by Lanskey et al. (2014) implicated changes to home and school environments, perceptions of the past and future and ability to adapt to the difficulties presented by paternal imprisonment as factors influencing children's well-being (e.g., Lanskey, Lösel, Markson, & Souza, 2014), suggesting that such factors need to be taken into account when striving to understand developmental processes (Bronfenbrenner, 1979). Using developmental psychopathology as the conceptual framework and adopting a resilience perspective, we argue that the impact of paternal imprisonment on children should be studied using a contextualized approach. Such research has implications for how to effectively support the positive adaptation of children living with this type of adversity.

Developmental psychopathology proposes an “organizational view of development in which multiple factors, or levels of a given factor, are considered in the context of one another, rather than in isolation” (Blandon, Calkins, Grimm, Keane, & O'Brien, 2010, p. 737). Accordingly, the context is a crucially important consideration (Cummings, Davies, & Campbell, 2000; Motti-Stefanidi, Asendorpf, & Masten, 2012; Sameroff, 2000). Resilience is a complex meta-theoretical construct (e.g., Cicchetti, 2010; Luthar, Cicchetti, & Becker, 2000; Masten, 2007; Rutter, 2012) and relates to “dynamic processes of ‘psychological functioning’ that foster greater positive and reduced negative outcomes in the face of relative adversity, both at the present time and in the future” (Cummings et al., 2000, p. 146). Resilience is influenced by dynamic, multi-level, social, psychological and biological processes that can vary across individuals, problem types, time and circumstances (Cicchetti, 2010; Lösel & Farrington, 2012; Masten, 2007; Rutter, 2012). Resilience has been defined and operationalized in different ways (e.g., Rutter, 1993). In the current study, we defined resilience as the absence of problems in a high-risk sample (Rutter, 1990).

Multiple contextual risk factors can have a negative influence on children's development (Flouri, 2008), including low parental educational achievement, parental mental illness, poverty, neighbourhood factors and parental substance abuse (Cummings et al., 2000; Luthar, 1999; Masten & Powell, 2003; Shaw, Cris, Schonberg, & Beck, 2004; Werner & Smith, 1992). Often prisoners' families are living with additional stressors that exacerbate the negative experience of paternal imprisonment (e.g., Geller, Garfinkel, Cooper, & Mincy, 2009). They are likely to be economically deprived, socially excluded and lacking support. They may also be vulnerable to stigmatization as a result of the imprisonment. For these reasons, prisoners' children are a unique at-risk group. These risk factors may affect development indirectly through the parents' well-being and parenting behaviour (Luthar, 1999) or directly through bullying and victimization. Risk factors have been shown to have a cumulative, dose–response relationship to children's behavioural outcomes (Atzaba-Poria, Pike, & Deater-Deckard, 2004; Deater-Deckard, Dodge, Bates, & Pettit, 1998; Van der Laan, Veenstra, Bogaerts, Verhulst, & Ormel, 2010) although cumulative contextual risk is an under-researched topic and has been the focus of conceptual debate (Flouri, 2008; Lösel & Bender, 2003).

Family relationships also have a significant impact on children's development. Lamb (2012) identified the relationships between children and parents/significant others and between parents/significant others as the most important social influences and attachment theory provides a theoretical explanation for their significance (e.g., Bowlby, 1953). Responsive and supportive parenting, accepting family environments and close parent–child relationships have been associated with behavioural competence (Collins, Harris, & Susman, 1995; Masten et al., 1999). Lamb (2012) noted that the security associated with attachment relationships may mediate the effect of parent–child relationships on child adjustment. Parental relationship conflict and harmony may affect children's emotional security and adjustment (e.g., Cummings et al., 2000).

Accordingly, positive and supportive family relationships can have protective or buffering effects for children in contexts of parental divorce, abuse, deprivation and cumulative stress (e.g., Masten & Powell, 2003; Masten & Shaffer, 2006; Neighbors, Forehand, & McVicar, 1993; Osborn, 1990; Pianta, Egeland, & Sroufe, 1990). This suggests that they are important for the development of resilience (Garmezy, 1985; Lösel & Bender, 2003). For example, good relationships between parents and children are associated with positive child behavioural outcomes in high-risk environments (e.g., Cummings et al., 2000). Similarly, emotionally responsive caregiving has been shown to moderate the impact of social and economic deprivation, high stress and abusive family relationships on broadly defined measures of positive behavioural, social and emotional functioning (Egeland, Carlson, & Sroufe, 1993).

The buffering effects of family variables have been found to vary by level of risk. For example, marital relationship quality protected against the emergence of antisocial behaviour in boys only in low risk conditions (Vanderbilt-Adriance & Shaw, 2008). Supportive family relationships moderated the association between exposure to violence, poverty and everyday stressors and internalizing behaviour for African-American children at low but not high levels of risk (Li, Nussbaum, & Maryse, 2007). This pattern was termed “overwhelming-risk” by Li et al. (p. 30) after Luthar, Cicchetti, and Becker's (2000) “protective-reactive” (p. 547) classification of moderation effects because protective influences were overwhelmed by risk factors. This concept is important insofar as it relativizes too optimistic views on resilience in cases where numerous stressors accumulate and leave not much “space” for protective influences (Lösel & Bender, 2003).

Theory and empirical evidence on developmental psychopathology, resilience and family relationships suggest that paternal imprisonment may be experienced by children in nuanced ways but prisoners' children have rarely been studied from that perspective. There have been no published prospective longitudinal studies on the impact of cumulative family risks and protective factors on behavioural outcomes with a specific focus on prisoners' children. Therefore, we integrated contextual influences to understand whether cumulative family risk factors (e.g., poverty, parental mental health problems) during the first year of life have a dose–response relationship to behavioural outcomes for male prisoners' children at age 9. In addition, we examined whether support, shared responsibility in the mother–father relationship and close mother–child relationships moderated the impact of cumulative risk on behavioural outcomes, thereby demonstrating supportive processes or resilience. The results could highlight the potential value of considering the impact of contextual risks as well as existing family strengths in policy and practice approaches to improving behavioural outcomes for prisoners' children.

## Method

### Design and sample

The data were obtained in the Fragile Families and Child Wellbeing (FFCW) study, which is following a stratified sample of almost 5000 children born between 1998 and 2000 to mostly unmarried parents in the USA (Reichman, Teitler, Garfinkel, & McLanahan, 2001). Both parents gave informed consent at the baseline survey. The mother and father were the main respondents in each wave (birth, years 1, 3, 5 and 9) and other informants, such as the focal child, primary caregivers and teachers, were interviewed in later waves. The present analyses used mother, father and child reported data from the birth (baseline), year one, age 3 and age 9 surveys.

We analysed a subsample of 801 children who had experienced paternal imprisonment between the calendar year of the age 3 survey and the date of the age 5 survey. Girls represented 43.2% of the sample. At baseline, mothers averaged 22.76 (SD = 5.22) and fathers 25.05 (SD = 6.45) years of age. Most parents were Black (63.3% mothers, 65.8% fathers), but many were Hispanic (20% mothers, 21.7% fathers), others White (14.2% mothers, 9.2% fathers).

### Instruments

#### Cumulative risk

Ten variables from the baseline and year one surveys were used to measure risk factors in the families' circumstances. The mothers' and fathers' highest level of education was indicated on the scale: 1 = graduate or professional school, 2 = college or technical, 3 = high school or equivalent or 4 = less than high school. Parents' depression was assessed using the Composite International Diagnostic Interview-Short Form (CIDI-SF; Kessler et al., 1998). Diagnostic criteria were met if respondents reported feeling depressed or that they had lost interest in pleasurable activities for at least half the day for the majority of days in a consecutive 2-week period in the last 12 months. The mothers' reports of household income were used to calculate the ratio of income to poverty thresholds as defined by the US Census Bureau. The mothers also described the family structure on the scale: 1 = married, 2 =  cohabiting, 3 = visiting, 4 = friends, 5 = hardly talk, 6 = never talk and 7 = father unknown. In addition, they rated the safety of the streets around their home at night on a scale of 1 = “very safe” to 4 = “very unsafe”. Both parents were asked: “In the past month, how many days did you have five or more drinks in one day?” and “In the past month did you use cocaine, crack, speed, LSD or heroin or any other kind of hard drug?” Alcohol and drug items were combined to indicate problematic alcohol or drug use. Fathers reported whether they had done paid work in the previous week. Variables were transformed into binary coded risk present/absent variables to calculate the total cumulative risk for each family. Table 1 shows each indicator, the data collection wave, the risk criterion that was used and the percentage of the sample reaching the risk threshold.

**Table 1:** Risk indicators for the cumulative risk measure

*Indicator*	*Time*	*Risk defined by*	*Percent of sample reaching risk threshold*
Mother education	Baseline	< high school education	45.8
Father education	Baseline	< high school education	49.5
Mother depression	Year one	Meets depression criteria	17.1
Father depression	Year one	Meets depression criteria	11.6
Poverty (mother report)	Baseline	Ratio of total household income to official poverty threshold < 99%	51.6
Parents relationship (mother report)	Baseline	Parents hardly talk, never talk or father unknown	8.6
Safe streets (mother report)	Baseline	Streets are unsafe or very unsafe	20.6
Mother alcohol and drugs	Year one	Consumed 5+ alcoholic drinks on 1+ days in the past month Used hard drugs in the last month	9.6
Father alcohol and drugs	Year one	Consumed 5+ alcoholic drinks on 1+ days in the past month Used hard drugs in the last month	23.4
Father employment	Baseline	No paid work in the last week	37.3

#### Family moderators (protective factors)

The measures of support, shared responsibility and mother–child closeness that were developed in the FFCW study were used as family moderators (protective factors).

#### *Support*

At year three, mothers rated six items about support in their relationship with the father on a scale of 1 = “never” to 3 = “often”. Mothers indicated whether the father “is fair and willing to compromise when you have a disagreement”, “expresses affection or love for you”, “insults or criticises your ideas” (reverse coded), “encourages you to do things that are important to you”, “listens when you need someone to talk to” and “really understands your hurts and joys”. Scores ranged from 6 to 18. The reliability of the scale was *α* = .86.

#### *Shared responsibility*

Also at year three, mothers rated four items about shared responsibility with the child's father on the scale of 1 = “never” to 4 = “always”: “How often does father look after child when you need to do things?”, “How often does he run errands for you?”, “How often does he fix things around your home?” and “How often does he take child places he/she needs to go?” Scores ranged from 4 to 16. The reliability of the scale was *α* = .91.

#### *Mother–child closeness*

The children reported at year nine how close they felt to their mothers. They responded on a scale of 0 = “never” to 3 = “always” to the four statements: “Your mom talks over important decisions with you”, “Your mom listens to your side of an argument”, “Your mom spends enough time with you” and “Your mom misses events or activities that are important to you” (reverse scored). Scores ranged from 0 to 12. The reliability of the items was *α* = .35. The low reliability could reflect the heterogeneous behaviours addressed and the focus on both facts and feelings. The items were combined on the basis of face validity.

The reliabilities of the family moderators with our subsample were similar to those in the whole study sample (*α* = .86 for support, *α* = .89 for shared responsibility and *α* = .38 for mother–child closeness). To create a measure of accumulated protective factors support, shared responsibility and mother–child closeness total scores were *z* transformed and summed.

#### *Child behaviour outcomes*

We used the mothers' year nine reports on the Child Behavior Checklist for ages 6–18 (CBCL; Achenbach, 1992) to measure the children's behavioural difficulties. Following Turney and Wildeman (2015), we used the 67 available items from the internalizing and externalizing behaviour subscales. Items were scored from 1 = “not true” to 3 = “very true/often true”. Behavioural difficulties scores ranged from 67 to 201. The reliability of the scale was *α* = .96. In addition, the children's year nine reports on 14 items from the internalizing and externalizing behaviour subscales of the Self-Description Questionnaire (SDQ; e.g., Marsh, Relich, & Smith, 1981) were used. The children responded 0 = “not at all true” to 3 = “very true” to statements such as “I feel angry when I have trouble learning something”, “I often feel lonely” and “I worry about having someone to play with”. Scores ranged from 0 to 42. The reliability of the scale was *α* = .84. We combined the CBCL and SDQ scores to provide a more comprehensive outcome measure that reflected responses from different informants. The CBCL and SDQ scores were *z* transformed and the mean of the scores was calculated.

#### Data analysis

Data on the cumulative risk indicators and moderator variables were missing at random (MAR; Little, 1988) and were imputed using SPSS version 21.^1^ Pooled estimates from 40 multiple imputed datasets were used. Individual cumulative risk and protective factor items were imputed and the total scores were calculated after the imputation. Although recommended (e.g., Allison, 2002), interaction terms were not included in the imputation model because this would have excluded data on individual risk items. A comparison of the different methods revealed similar results. Table 2 shows the means and standard deviations of total cumulative risk and moderator variables before and after imputation. Outcome data were not imputed.

**Table 2:** Original and imputed descriptive statistics for cumulative risk and moderator variables

	*Original Mean (SD)*	*Imputed Mean (SD)*
Cumulative risk	2.68 (1.48) (Med = 3)	3.01 (1.58) (Med = 3)
Support	13.09 (3.45)	13.06 (3.35)
Shared responsibility	9.10 (4.57)	9.12 (4.27)
Mother–child closeness	8.12 (2.42)	7.79 (2.33)

Bivariate correlations between cumulative risk, behavioural outcomes and moderator variables were examined. To investigate moderator effects, we carried out hierarchical multiple regression analyses. The potential protective factors were mean-centered before the analysis. Total cumulative risk was first entered into the regression model, then the moderator variable and finally the product term of cumulative risk and the moderator (Hayes, 2013).

## Results

### Descriptive and bivariate analyses

For the outcomes, average scores were *M* = 80.47 (*SD* = 15.17) for the CBCL and *M* = 16.73 (*SD* = 8.99) for the SDQ.

As expected, cumulative risk was negatively correlated with the potential family protective factors and the latter were negatively correlated with the child behaviour outcomes (Table 3). Cumulative risk significantly predicted behavioural outcomes at age 9: CBCL, *r* = .21, *p* < .01; SDQ, *r* = .13, *p* < .01; and the combined measure, *r* = .19, *p* < .01.

**Table 3:** Cumulative risk correlated with behavioural outcomes and family moderators

*Family moderators*	*Cumulative risk*	*CBCL (MI)*	*SDQ (CI)*	*CBCL and SDQ combined*
Support	− 0.08^*^	− 0.09	− 0.05	− 0.09^*^
Shared responsibility	− 0.09^*^	− 0.04	− 0.08^*^	− 0.09^*^
Mother–child closeness	− 0.05	− 0.08	− 0.13^* *^	− 0.12^* *^

^*^ *p* < .05, ^* *^ *p* < .01, two-tailed, *n* = 531–801, variation due to missing data on outcome variables, MI =  mother informant, CI = child informant.

*Interaction analyses*: There was statistically significant evidence of moderation by mother–child closeness for the SDQ and combined outcome measures, and by accumulated protective factors for the combined outcome, although the effects were weak. The results are presented in Table 4.

**Table 4:** Moderation results

		*CBCL*	*SDQ*	*CBCL and SDQ combined*
*Support*		Coeff. (*SE*)	Coeff. (*SE*)	Coeff. (*SE*)
Intercept	*b*_*0*_	74.54^* * *^ (1.49)	14.49^* * *^ (0.80)	− 0.29^* * *^ (0.07)
Cumulative risk	*b*_1_	1.98^* * *^ (0.45)	0.75^* *^ (0.24)	0.10^* * *^ (0.02)
Support	*b*_*2*_	− 0.54 (0.45)	− 0.29 (0.25)	− 0.04^a^ (0.02)
Cumulative risk × support	*b*_*3*_	0.08 (0.15)	0.06 (0.07)	0.01 (0.01)
*R*^2^ (overall model) =		0.050	0.021	0.046
*Shared responsibility*				
Intercept	*b*_*0*_	74.46^* * *^ (1.48)	14.56^* * *^ (0.79)	− 0.28^* * *^ (0.07)
Cumulative risk	*b*_1_	2.01^* * *^ (0.44)	0.72^* *^ (0.24)	0.10^* * *^ (0.02)
Shared resp.	*b*_*2*_	− 0.25 (0.37)	− 0.12 (0.20)	− 0.02 (0.02)
Cumulative risk × Shared resp.	*b*_*3*_	0.06 (0.12)	− 0.01 (0.06)	0.00 (0.01)
*R*^2^ (overall model) =		0.046	0.024	0.042
*Mother–child closeness*				
Intercept	*b*_*0*_	74.55^* * *^ (1.47)	14.60^* * *^ (0.79)	− 0.28^* * *^ (0.07)
Cumulative risk	*b*_1_	1.98^* * *^ (0.44)	0.72^* *^ (0.23)	0.10^* * *^ (0.02)
Mother–child closeness	*b*_*2*_	− 1.03^a^ (0.62)	− 1.01^* *^ (0.33)	− 0.10^* *^ (0.03)
Cumulative risk x mother–child closeness	*b*_*3*_	0.18 (0.18)	0.17^a^ (0.10)	0.02^*^ (0.01)
*R*^2^ (overall model) =		0.052	0.039	0.059
*Accumulated protective factors (APF)*				
Intercept	*b*_*0*_	74.65^* * *^ (1.48)	14.66^* * *^ (0.79)	− 0.27^* * *^ (0.07)
Cumulative risk	*b*_1_	1.96^* * *^ (0.45)	0.70^* *^ (0.24)	0.09^* * *^ (0.02)
APF	*b*_*2*_	− 1.18^a^ (0.71)	− 0.90^*^ (0.39)	− 0.10^* *^ (0.04)
Cumulative risk × APF	*b*_*3*_	0.21 (0.22)	0.13 (0.12)	0.02 ^a^ (0.01)
*R*^2^ (overall model) =		0.053	0.034	0.058

*p* ≤ .10, ^*^ *p* < .05, ^* *^ *p* < .01, ^* * *^ *p* < .001, two-tailed, n = 531–801, variation due to missing data on outcome variables.

**Figure 1:**
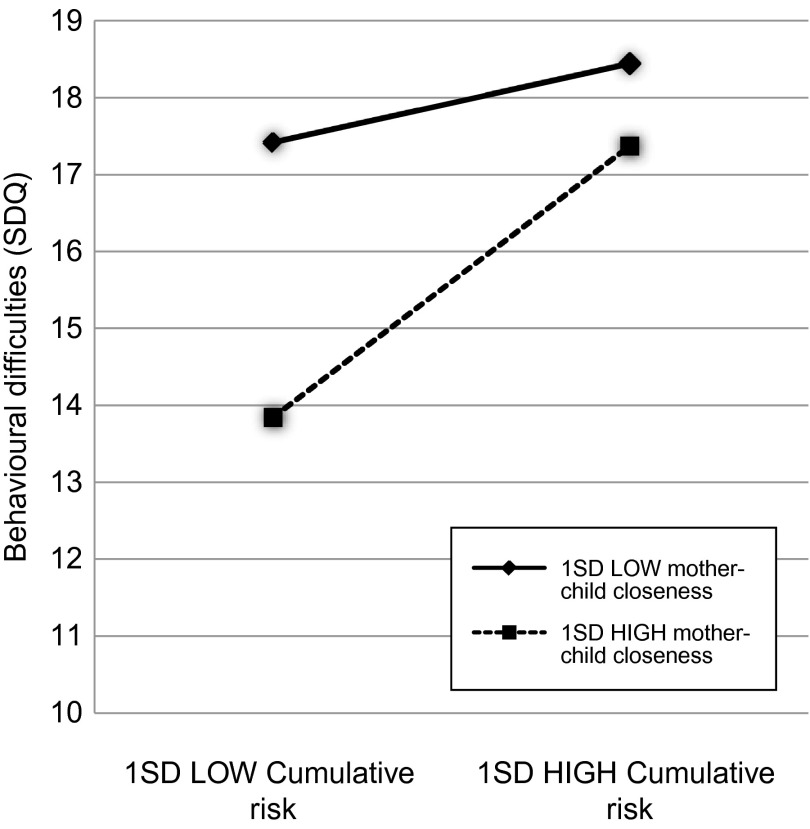
Graph showing interaction among cumulative risk, mother–child closeness and SDQ outcome

**Figure 2:**
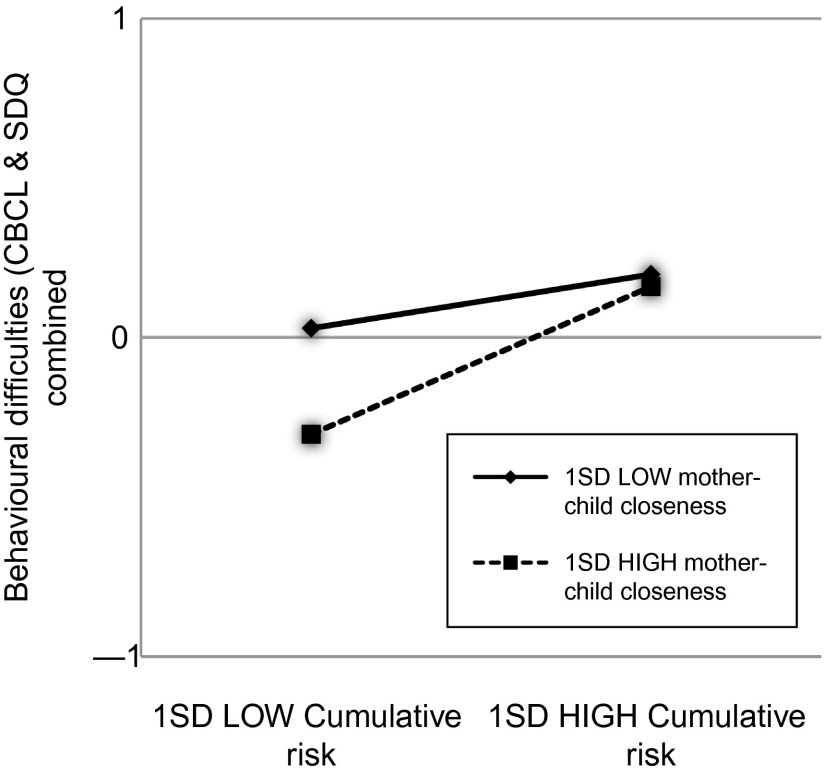
Graph showing interaction among cumulative risk, mother–child closeness and CBCL and SDQ combined outcome

**Figure 3:**
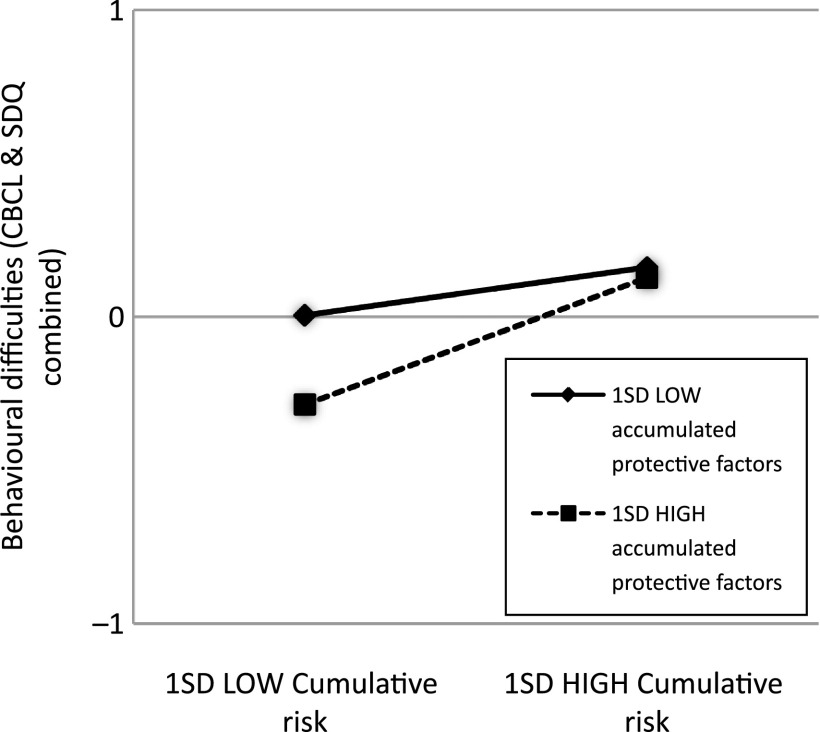
Graph showing interaction among cumulative risk, accumulated protective factors and CBCL and SDQ combined outcome

To plot the statistically significant interactions, values one SD below and above the mean for cumulative risk (1.43 and 4.59, respectively) and the moderators were used to represent *X* and *M* in the regression equation. For all models, problem scores on the outcome measures were lowest when the moderator was “high” and cumulative risk was “low”. To illustrate, behaviour was more positive for children with “low” cumulative risk and “high” mother–child closeness than for children with “low” cumulative risk and “low” mother–child closeness. However, at “high” cumulative risk, there was no difference in behaviour between children with “low” and “high” mother–child closeness scores (Figures 1 and 2). The same pattern was observed with accumulated protective factors and the combined behaviour outcome (Figure 3).

## Discussion

This study examined the importance of family context for prisoners' children's behavioural development. It investigated whether cumulative risks around the first year of life predicted behavioural outcomes in middle childhood. In a more differentiated approach, it also investigated whether within-family factors had a protective function and contributed to resilience. In particular, supportive parental relationships, shared responsibility and mother–child closeness were considered as potential moderators of the relationship between cumulative risks and behavioural outcomes. Cumulative risk was significantly and positively associated with behavioural difficulties, but the effect sizes were small. Results were clearest for the combined behavioural outcome measure but no strong support for moderation was found. Although statistically significant, the moderation by mother–child closeness for child reported and combined behavioural adjustment measures was weak. Support and shared responsibility did not moderate behavioural outcomes. Weak evidence of moderation by accumulated protective factors was also found for mother and child combined reports. The data showed that high levels of mother–child closeness were protective against problem behaviour in the context of low cumulative risk. Thus, for children affected by paternal imprisonment in low family risk environments, the presence of positive family relationships appeared to provide a protective effect that somewhat diminished the negative impact of risk on positive behavioural development. The findings point to protective effects of mother–child relationships as well as the possible value of accumulated family relationship factors. There were no protective effects at high levels of cumulative risk.

Although our bivariate correlations were modest, the finding that cumulative risk predicted children's later behavioural outcomes is in agreement with results from general population samples (e.g., Deater-Deckard et al., 1998; Van der Laan et al., 2010). The findings support the notion that prisoners' children are not a homogeneous group and that their outcomes are influenced by other risk factors beyond parental imprisonment. This finding suggests dose–response effects of adversity exposure. Moderation by family relationship factors on behaviour problems at lower levels of cumulative risk has also been reported in other studies (e.g., Li et al., 2007; Vanderbilt-Adriance & Shaw, 2008) highlighting the importance of these protective factors in certain risk contexts. For families with few risk factors, close mother–child relationships could have enhanced the emotional security of the children, having a positive impact on behaviour. While not individually protective, the accumulation of benefits from support and shared responsibility could have a positive impact on family environment, the availability of resources, parenting and children's emotional security (e.g., Cummings et al., 2000; Lamb, 2012).

However, our results do not strongly support protective mechanisms from functional family characteristics. Even accumulated potentially protective factors did not have a substantial impact. Such findings are somewhat inconsistent with evidence regarding protective factors and resilience in children experiencing other adversities such as parental divorce, abuse and socioeconomic disadvantage (e.g., Osborn, 1990; Masten & Powell, 2003; Masten & Shaffer, 2006; Neighbors, Forehand, & McVicar, 1993; Pianta, Egeland, & Sroufe, 1990). There are several possible reasons for this divergence. The correlations between the risk factors in early childhood and the child behaviour outcomes were rather low, so there was not much ‘space’ for buffering or moderating effects. Although we included important risk factors such as poverty, low parental education, substance misuse and mental health problems in our cumulative index, its predictive validity was small. This is in agreement with risk assessment research showing low correlations between very early risk factors and behavioural outcomes after longer time intervals (e.g., Hawkins et al., 1998; Lösel & Bender, 2006). Such findings illustrate important processes of flexibility and multifinality in behavioural development. Although studying development over the longer term is essential for understanding dynamic processes of resilience (Luthar et al., 2000), the long period of time covered from child birth to age 9 may partially explain the weak associations. Dose–response effects that diminish over time have been explained in terms of recovery from adversity (e.g., Masten & Osofsky, 2010). Of the moderators we analysed, child-reported mother–child closeness at year nine provided the strongest associations with outcomes, suggesting that protective factors measured temporally closer to outcomes have more influence than those measured temporally further away. This finding fits with the above-mentioned interpretations.

The absence of strong protective influences of functional family factors may also be indicative of Luthar et al.'s (2000) “protective-reactive” and Li et al.'s (2007) “overwhelming risk pattern”. In our high-risk sample with relatively low variability in risk, one can assume that there were too many problems for substantial protective influences to be identified, even at lower levels of risk, and therefore any protective influences were overwhelmed by risk factors. Prisoners' families are likely to have many problems and be among the most disadvantaged groups in society. In our sample, around half the families reached the risk thresholds for the parents' education and poverty indicators. Therefore, low risk in our sample is not comparable to low risk in a “normal” population sample. Even low risk families are at rather high risk for child developmental problems, but are at lower risk in comparison to others in the sample. The difficulties in identifying protective effects with high-risk samples have been noted by other researchers (e.g., Loeber & Farrington, 2012; Vanderbilt-Adriance & Shaw, 2008).

Some limitations to this study should be mentioned. We did not examine potentially protective individual characteristics (Li et al., 2007; Masten & Powell, 2003; Masten & Shaffer, 2006; Motti-Stefanidi et al., 2012; Werner & Smith, 1992), specificity of risks and outcomes (Masten & Powell, 2003), non-linear relationships or the stability of resilience processes (Luthar et al., 2000). Another limit is our main reliance on information from the mothers because not enough of the fathers provided data consistently. As in other areas of research, perspectives of different family members are also highly important for studies on parental imprisonment (Harris, Graham, & Carpenter, 2010; Souza, Lösel, Markson, & Lanskey, 2013). Because of non-random attrition, families affected by imprisonment may be underrepresented in later study waves which limit the generalizability of the findings (Schwartz-Soicher, Geller, & Garfinkel, 2011). It should also be mentioned that there is no established method for creating a cumulative risk index and no agreement about what risk factors to include. Perhaps a more domain-specific and theory-based combination of risks may reveal stronger effects (e.g., Flouri, 2008). Finally, our investigation of protective factors was limited to using short scales and the reliability of the mother–child closeness measure was low, although it possessed face validity (e.g., Card & Barnett, 2015).

Notwithstanding these limitations, this study represented a timely investigation of the under-researched topics of cumulative risk and protection with a large number of hard-to-reach families experiencing the specific adversity of paternal imprisonment. In principle, the findings support a cumulative risk and protective factors approach for the study of this group. They suggest that protective processes may operate in areas that are possible to target through support and intervention. A tentative policy implication is that better behavioural outcomes for prisoners' children may be achieved if family risk factors are reduced and protective factors are increased. However, more research is needed to explore processes of risk and resilience in prisoners' families. For example, the interaction between biological and social factors in relation to protective effects for behavioural development has been studied very little (Lösel & Bender, 2003).

Although the findings do not strongly inform our understanding of contextual family influences on resilience processes in children of prisoners, in our high risk sample small effects are worth noting; they do not suggest that contextual environments do not matter. Instead, they underscore how difficult it is to study prisoners' families. An additional important aim for research remains to identify effective policy to support the positive development of children experiencing paternal imprisonment in cases where the level of risks is very high and may overwhelm “natural” protective resources. This relates to several areas of further study: examining temporal relationships among risks, protective influences and outcomes to establish whether there are “sensitive periods” at which effects can be revealed; studying individual characteristics of prisoners' children to identify protective resources; and integrating protective factors from different domains to reveal effects in conditions of high adversity.

## Disclosure statement

No potential conflict of interest was reported by the authors.

## Funding

This work was supported by the Economic and Social Research Council. The authors thank the organizers of the Fragile Families and Child Wellbeing Study Summer Data Workshop 2013 for their introduction to the data-set and Steve Lainé for his technical support.

## Footnotes

^1^ On most variables, no more than 15% of the data were missing. Exceptions were father depression (33.2%), father alcohol (33.8%), father drugs (33.2%), father employment (23.7%), shared responsibility (21.7%) and mother–child closeness (26.5%).
